# The SOFG Anatomy Entry List (SAEL): An Annotation Tool for Functional Genomics Data

**DOI:** 10.1002/cfg.434

**Published:** 2004

**Authors:** Helen Parkinson, Stuart Aitken, Richard A. Baldock, Jonathan B. L. Bard, Albert Burger, Terry F. Hayamizu, Alan Rector, Martin Ringwald, Jeremy Rogers, Cornelius Rosse, Christian J. Stoeckert, Duncan Davidson

**Affiliations:** 1 European Bioinformatics Institute Wellcome Trust Genome Campus Hinxton, Cambridge CB10 1SD UK; 2 Artificial Intelligence Applications Institute School of Informatics University of Edinburgh Crichton Street Edinburgh EH8 9LE UK; 3 MRC Human Genetics Unit Western General Hospital Crewe Road Edinburgh EH4 2XU UK; 4 Wolfson Laboratory Medical School University of Edinburgh Edinburgh EH8 9XD UK; 5 School of Mathematics and Computer Sciences Heriot–Watt University Riccarton, Edinburgh EH14 4AS UK; 6 The Jackson Laboratory 600 Main Street Bar Harbor ME 04609 USA; 7 Kilburn Building University of Manchester Oxford Road Manchester M13 9PL UK; 8 Department of Biological Structure and Department of Medical Education and Biomedical Informatics School of Medicine University of Washington Seattle WA USA; 9 Department of Genetics Center for Bioinformatics University of Pennsylvania 423 Guardian Drive Philadelphia PA 19104 USA

## Abstract

A great deal of data in functional genomics studies needs to be annotated with low-resolution anatomical terms. For example, gene expression assays based on manually dissected samples (microarray, SAGE, etc.) need high-level anatomical terms to describe sample origin. First-pass annotation in high-throughput assays (e.g. large-scale *in situ* gene expression screens or phenotype screens) and bibliographic applications, such as selection of keywords, would also benefit from a minimum set of standard anatomical terms. Although only simple terms are required, the researcher faces serious practical problems of inconsistency and confusion, given the different aims and the range of complexity of existing anatomy ontologies. A Standards and Ontologies for Functional Genomics (SOFG) group therefore initiated discussions between several of the major anatomical ontologies for higher vertebrates. As we report here, one result of these discussions is a simple, accessible, controlled vocabulary of gross anatomical terms, the SOFG Anatomy Entry List (SAEL). The SAEL is available from http://www.sofg.org and is intended as a resource for biologists, curators, bioinformaticians and developers of software supporting functional genomics. It can be used directly for annotation in the contexts described above. Importantly, each term is linked to the corresponding term in each of the major anatomy ontologies. Where the simple list does not provide enough detail or sophistication, therefore, the researcher can use the SAEL to choose the appropriate ontology and move directly to the relevant term as an entry point. The SAEL links will also be used to support computational access to the respective ontologies.


**Comparative and Functional Genomics**
*Comp Funct Genom* 2004; **5**: 521–527.
Published online in Wiley InterScience (www.interscience.wiley.com). **DOI:** 10.1002/cfg.434

**Conference Paper**

### The SOFG Anatomy Entry List (SAEL): an annotation tool for functional genomics data

Report of a Workshop on Integration of Anatomy Ontologies, held at the MRC Human Genetics Unit, Edinburgh, 7–8 April 2004

Helen Parkinson[1], Stuart Aitken[2], Richard A. Baldock[3], Jonathan B. L. Bard[4], Albert Burger[3,5], Terry F. Hayamizu[6], Alan Rector[7], Martin Ringwald[6], Jeremy Rogers[7], Cornelius Rosse[8], Christian J. Stoeckert Jr[9] and Duncan Davidson[3]*

[1] *European Bioinformatics Institute, Wellcome Trust Genome Campus, Hinxton, Cambridge CB10 1SD, UK*
[2] *Artificial Intelligence Applications Institute, School of Informatics, University of Edinburgh, Crichton Street, Edinburgh EH8 9LE, UK*
[3] *MRC Human Genetics Unit, Western General Hospital, Crewe Road, Edinburgh EH4 2XU, UK*
[4] *Wolfson Laboratory, Medical School, University of Edinburgh, Edinburgh EH8 9XD, UK*
[5] *School of Mathematics and Computer Sciences, Heriot–Watt University, Riccarton, Edinburgh EH14 4AS, UK*
[6] *The Jackson Laboratory, 600 Main Street, Bar Harbor, ME 04609, USA*
[7] *Kilburn Building, University of Manchester, Oxford Road, Manchester M13 9PL, UK*
[8] *Department of Biological Structure and Department of Medical Education and Biomedical Informatics, School of Medicine, University of Washington, Seattle, WA, USA*
[9] *Department of Genetics, Center for Bioinformatics, University of Pennsylvania, 423 Guardian Drive, Philadelphia, PA 19104, USA*

**Correspondence to:*
*Duncan Davidson, MRC Human Genetics Unit, Western General Hospital, Crewe Road, Edinburgh EH4 2XU, UK.*
*E-mail:*
*Duncan.Davidson@hgu.mrc.ac.uk*

Received: 23 October 2004
Revised: 1 November 2004
Accepted: 2 November 2004

### Abstract

**A great deal of data in functional genomics studies needs to be annotated with low-resolution anatomical terms. For example, gene expression assays based on manually dissected samples (microarray, SAGE, etc.) need high-level anatomical terms to describe sample origin. First-pass annotation in high-throughput assays (e.g. large-scale *in situ* gene expression screens or phenotype screens) and bibliographic applications, such as selection of keywords, would also benefit from a minimum set of standard anatomical terms. Although only simple terms are required, the researcher faces serious practical problems of inconsistency and confusion, given the different aims and the range of complexity of existing anatomy ontologies. A Standards and Ontologies for Functional Genomics (SOFG) group therefore initiated discussions between several of the major anatomical ontologies for higher vertebrates. As we report here, one result of these discussions is a simple, accessible, controlled vocabulary of gross anatomical terms, the SOFG Anatomy Entry List (SAEL). The SAEL is available from http://www.sofg.org and is intended as a resource for biologists, curators, bioinformaticians and developers of software supporting functional genomics. It can be used directly for annotation in the contexts described above. Importantly, each term is linked to the corresponding term in each of the major anatomy ontologies. Where the simple list does not provide enough detail or sophistication, therefore, the researcher can use the SAEL to choose the appropriate ontology and move directly to the relevant term as an entry point. The SAEL links will also be used to support computational access to the respective ontologies. Copyright © 2004 John Wiley & Sons, Ltd.**

**Keywords:** **anatomy; annotation; ontology; microarray; high-throughput; phenotype description; bibliographic keywords**

Copyright © 2004 John Wiley & Sons, Ltd.

### Introduction

This paper addresses the problem of annotating gene function-related data with anatomical terms. Our focus here is on simple annotation at low anatomical resolution, such as is required to describe the origin of tissue used for microarray or other sample-based analyses. A similar requirement exists for simple, first-pass annotation in high-throughput screens of *in situ* gene expression patterns or of parts affected in mutant phenotypes. A simple, standard annotation scheme would also find application in selecting keywords, or terms in tables of results, providing support for automated bibliographic data retrieval.

At first sight, it seems trivial to annotate the source of samples for microarray experiments as 'heart', 'kidney', etc. However, even cursory examination of the literature is sufficient to show that a simple 'free text' approach leads to inconsistencies. These curtail the usefulness of annotations for database purposes and for information retrieval from the literature. The solution is to use a controlled vocabulary or ontology. The long-running study of anatomy and the need for common annotation in biology and medicine have resulted in a proliferation of biomedical ontologies built for different purposes, using different knowledge representation tools and often very rich in terms, structure and relationship types. Anatomy components in biomedical ontologies serve varied purposes, e.g. descriptions of medical procedures in GALEN (**www.opengalen.org**), or descriptions of traits or phenotypes. The multiple anatomy ontologies often contain non-orthogonal concepts, although these are often defined and structured differently within each ontology. For example, the Foundational Model of Anatomy (FMA; Rosse and Mejino, 2003), which takes a structural view of anatomy, contains the concept 'liver' defined in free text as 'Lobular organ the parenchyma of which consists of lobules which communicate with the biliary tree'. 'Liver' is also defined by its attributes, including: 'member-of', 'bounded-by', 'component-of', 'adjacency', etc. If we consider 'liver' in the Mouse Developmental Anatomy Dictionary (Bard *et al.*, 1998), which has a developmental view, the 'liver' is 'part-of ' the 'liver and biliary system' and developmental stage information is provided. The level of detail provided by these ontologies is variable and the purposes of the ontologies are clearly different, although both contain the concept 'liver'.

Selection of anatomical terms for functional genomics annotation therefore requires some knowledge of where to look, some information on the purpose and scope of the ontology queried, and an ability to critically assess whether the term returned is accurate. These tasks may be intuitive for the average scientist who has some notion of the concept of each term. However, in a high-throughput situation it is time consuming to query large and complex ontologies directly and, in our experience, many scientists simply annotate with free text. This causes problems for data exchange and query in functional genomics.

From the viewpoint of the biologist who wishes to annotate data with anatomical terms, there are two primary requirements. The first is a standard, high-level vocabulary that is simple and easy to use but nevertheless carries the authority of the established anatomical reference sources. The second requirement is a single point of entry to the available ontology resources in situations that require a more complex anatomical nomenclature. This entry point should enable the biologist to choose the appropriate ontology and to quickly find the appropriate terms.

In this paper, we report the results of initial discussions between several of the major anatomical ontologies for higher vertebrates. These discussions were initiated following a conference on Standards and Ontologies for Functional Genomics (SOFG, Hinxton, UK 2002) and continued at the Workshop described below. They are aimed at practical moves towards integration. As a first step, we propose a standard anatomy entry list (the SAEL), which aims to meet the two requirements described above.

### The standard anatomy entry list (SAEL)

Following the SOFG 2002 conference, a website was established listing many of the major ontologies and resources for human and mouse anatomy (**http://www.sofg.org**). The website provides URL links and contact names for the ontologies and outlines their purpose and structure. In April 2004, a 2-day workshop was organized at the MRC Human Genetics Unit in Edinburgh to examine practical ways to integrate information in different ontologies. In particular, two

Copyright © 2004 John Wiley & Sons, Ltd. 

questions were considered: (a) can a 'core anatomy list' be produced for functional genomics applications, specifically microarray experiments? and (b) if so, what are the use cases, limitations, and build criteria? The small group of workshop participants represented the following anatomy ontologies: Open Galen (**www.opengalen.org**), the FMA (**http://fma.biostr.washington.edu/**; Rosse and Mejino, 2003), the ontology of human developmental anatomy, HUMAT (Hunter *et al.*, 2003), the Edinburgh Mouse Atlas Project (EMAP) Mouse Developmental Anatomical Dictionary (**http://genex.hgu.mrc.ac.uk/Databases/Anatomy/**; Bard *et al.*, 1998), the Adult Mouse Anatomical Dictionary (**http://www.informatics.jax.org/searches/AMA_form.shtml**) and the CIBL Controlled Vocabulary for Anatomy (**http://www.cbil.upenn.edu/anatomy.php3**). Also represented were users of anatomy ontologies: ArrayExpress (Brazma *et al.*, 2003); the RNA Abundance Database, RAD (Manduchi *et al.*, 2004); the Gene Expression Database, GXD (**http://www.informatics.jax.org/mgihome/GXD/aboutGXD.shtml;** Hill *et al.* 2004) and the Edinburgh Mouse Atlas of Gene Expression, EMAGE (**http://genex.hgu.mrc.ac.uk**). The group also included expert logicians and computer scientists. A more detailed report from the workshop is available at the SOFG website (**http://www.sofg.org**). The efforts of this workshop and subsequent intensive e-mail discussions produced a draft list of anatomy terms, the SOFG Anatomy Entry List (SAEL), described below, which is being mapped to the different ontologies represented at the workshop. Other ontologies represented on the SOFG website are also contributing and providing mappings to the list. We are now moving to wider consultation, testing and use with the eventual aim of wide contribution and 'ownership' of this simple resource.

### Purpose of the SAEL

The purpose of the SAEL is to provide a manageable list of anatomical terms that can be used to annotate gene function data at low anatomical resolution. Importantly, this list also provides an entry point for access to the major, freely available anatomy ontologies for human and rodents. This access aims to be both visual, via graphical user interfaces, and computational, to facilitate automatic data retrieval. The SAEL therefore aims to be a community resource for biologists, curators, informaticians and developers of software supporting functional genomics.

### Design and methods

### The SAEL is a controlled vocabulary of terms referring to gross anatomical structures

The list is envisaged as comprising 100–150 terms, each with a unique identity number. It has sufficient resolution to distinguish samples obtained by gross dissection, e.g. for microarray experiments, and to broadly classify expression or mutant phenotypes, e.g. in high-throughput screens. Even a list of such low-resolution anatomical terms has wide application and can serve to annotate a very large amount of data.

### The SAEL is not an ontology

The SAEL is simply an unstructured list of terms. It is, of course, possible to subdivide the list with headings such as 'Developmental structures' or 'Organ systems' for purposes of presentation, but such headings are not part of the list and have no identity numbers. SAEL terms are intentionally not defined, as definitions tie terms to species and 'views' of anatomy that the authors wish to avoid and the list contains no information about relationships between anatomical structures. The advantage of this approach is that it removes from immediate concern differences in the way the specialized ontologies view definitions and relationships (e.g. 'part-of' and 'is-a' relationships), while providing links to this information for situations where it is needed.

### Each term in the SAEL is mapped to the corresponding terms in the different participating ontologies

On their own, the SAEL terms are clearly not sufficient for all annotation applications, e.g. they do not have sufficient resolution to describe the origins of samples dissected by laser capture microscopy or to describe *in situ* gene expression patterns where interest is focused on one particular organ. Access to finer-grain, more sophisticated resources is required. By mapping SAEL terms to the participating ontologies, the simple list provides a means to find out what ontologies are available, go directly to the relevant part in any one of these, compare

Copyright © 2004 John Wiley & Sons, Ltd.

them and choose the resource that best suits a particular need.

To map the SAEL to a target ontology, each SAEL term (and its unique identifier) is matched to the appropriate single term (and its unique identifier) in the ontology. These mappings are being made manually by experts from the groups responsible for building and maintaining the target ontologies. This is done using the COBrA graphical interface, an ontology-linking tool developed by the Cross-Species Anatomy Network Project, XSPAN (**www.xspan.org**). COBrA reads DAGEdit flat file format, GO XML/RDF, GO RDFS and OWL formats and creates an OWL format mapping file. Null mappings will be recorded as such (e.g. from the SAEL term 'tail' to human anatomy ontologies).

### Attributes of objects in the target ontologies will be returned by queries using the corresponding SAEL term

A biologist or database curator will be able to move directly from any SAEL term to the corresponding location in a SAEL-mapped ontology resource. In addition, it will be possible to use SAEL terms to make simple queries across SAEL-mapped ontologies in order to support automatic data retrieval in bioinformatics applications. An example would be the use of a SAEL term (e.g. 'pancreas; SAEL:78') to interrogate datasets annotated using different ontologies.

The Workshop considered the set of attributes that might be returned by a web services query when using a SAEL term to query a SAEL-mapped ontology resource. A provisional list of attributes is shown in Table 1. It is intended that source ontologies will provide a web service supporting the attribute list. These attributes, taken together, may constitute a Web Services Description Language (WSDL) description for anatomy ontologies.

### The current list is specifically focused on rodent and human anatomy

In mouse, rat and human anatomy, the equivalence of terms and the homology of the structures to which they refer are generally not contentious. Extension to other vertebrates will have to deal with more contentious issues of homology (see e.g. Hall, 1995). We view such extension of the

**Table 1.** Definition of example attributes returned from target ontologies by queries using terms on the SOFG Anatomy Entry List (SAEL)

| Attribute | Allowed values | Definition |
|---|---|---|
| dev_stage | n/a | The developmental stage(s) that is annotated to the anatomical entity. Note that general associations, such as those implied from the anatomical resource being developed for the 'adult' of the species, are not sufficient |
| is_tissue | yes, no, nil | The anatomical entity is explicitly categorized as a tissue, e.g. the anatomical entity is within a strict 'is-a' hierarchy for tissue |
| is_cell_type | yes, no, nil | The anatomical entity is explicitly categorized as a type of cell, e.g. the anatomical entity is within a strict 'is-a' hierarchy for cell type |
| is_organ | yes, no, nil | The anatomical entity is explicitly categorized as an organ, e.g. the anatomical entity is within a strict 'is-a' hierarchy for organ |
| is_system | yes, no, nil | The anatomical entity is explicitly categorized as a system, e.g. the anatomical entity is within a strict 'is-a' hierarchy for system |
| superclass | n/a | The immediate class(es) above the anatomical entity in an 'is-a' relationship |
| subclass | n/a | The immediate class(es) below the anatomical entity in an 'is-a' relationship |
| Part | n/a | The immediate class(es) below the anatomical entity in a 'part-of' relationship |
| part_of | n/a | The immediate class(es) above the anatomical entity in a 'part-of' relationship |
| uri | n/a | The uniform resource identifier for the anatomical entity in the anatomical resource. A URL is a common type of URI. The URI should provide a pointer directly to information on the anatomical entity in the anatomical resource. This may be in the form of a cgi command |
| definition | n/a | The definition provided by the targeted resource for the anatomical entity |
| authority | n/a | The source of the information provided at the target resource for the anatomical entity. The source may be a person, an organization, or a literature citation |
| history | n/a | The data provenance of the anatomical entity. Usually, this is the last modification time and date at the anatomical resource for information directly associated with the anatomical entity |
| name: | n/a | The name given to the anatomical entity by the anatomical resource |
| synonym | n/a | The alternative name(s) given to the anatomical entity by the anatomical resource |

Copyright © 2004 John Wiley & Sons, Ltd. 

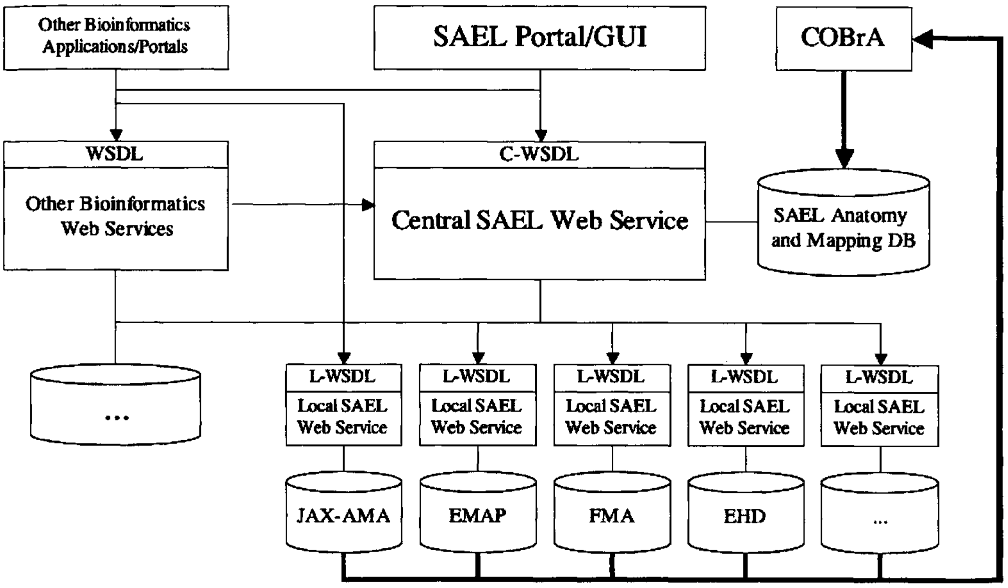

**Figure 1.** SAEL web service architecture

SAEL as proceeding later in collaboration with comparative anatomists and specialist ontology projects that are currently dealing with these issues (e.g. XSPAN; **www.xspan.org**).

#### Implementation: SAEL Software Architecture

The SAEL and mappings to individual anatomy ontologies will be made publicly accessible in simple downloadable form as well as through a programmatic interface and a graphical user interface. Figure 1 shows the preliminary software architecture in support of SAEL. The SAEL Anatomy and Mapping database will hold representations of the SAEL list of anatomical entities, parts of the target ontologies and mappings between SAEL entities and the ontologies. The COBrA tool is used to capture the mappings and make them available to the SAEL database.

Each participating anatomy resource will provide a web service interface to its ontology, supporting a simple query mechanism based on the attribute list described in Table 1. Queries based on those attributes that involve more than one ontology will be supported by the Central SAEL Web Service. The corresponding central and local WSDL descriptions (C-WSDL and L-WSDL) will define the exact access details to these services. The SAEL Portal will provide a graphical user interface for researchers to look up the mappings between the SAEL list of anatomical entities and the target ontologies.

We expect that the SAEL service will become part of a wider network of bioinformatics resources that links anatomy ontologies with other ontologies, databases and computational services.

Most of the computational infrastructure — the Central Web Service, the Anatomy and Mapping DB, the Portal and COBrA — required to deliver SAEL services has already been developed as part of the XSPAN project and will be used for SAEL. Implementation will be carried out in two phases: the SAEL portal will be implemented first, then local web services.

### The SAEL list

The current version (1.1) of the SAEL is freely available at the SOFG website (**http://www.sofg.org**) and may be downloaded in OBO format or as plain text. The list will be modified and extended as necessary, with careful versioning, and within the limits of the requirement that it be manageable. New terms may be proposed via the Microarray Gene Expression Data Society (MGED) Ontology Tracker. Please mail: **mged-ontologies@lists.sourceforge.net** with feedback. The SAEL is currently maintained by the MGED Ontology group, with anatomical curation by Jackson Laboratory GXD database curator Terry

Copyright © 2004 John Wiley & Sons, Ltd.

Hayamizu. Mappings between the SAEL and participating anatomy ontologies are also available from the SOFG website.

### Gene–function databases that will use the SAEL

The SAEL will be used by the following gene–function related databases: RAD, ArrayExpress, MIAMExpress. In most cases, annotation in these databases will not, of course, be confined to SAEL terms, but the ontologies employed will be mapped to the SAEL. The MIAMExpress–ArrayExpress data capture tool uses the SAEL. Data in ArrayExpress will be mapped to SAEL and future submissions will use the SAEL. The SAEL will be included in MGED Ontology v1.2 (**http://mged.sourceforge.net/ontologies/index.php**).

### Discussion

The current status of the work is that version 1.1 of the SAEL is available. Mappings have been made to the FMA and the Jackson Laboratory Adult Mouse ontology and are in progress for other participating ontologies (these will be available by December 2004). The interfaces and the web services portal are being developed. Current SAEL resources are available at **http://www.sofg.org**

In a preliminary survey, the content of the SAEL was tested for correspondence to annotations of sample-based data in the following gene expression databases: ArrayExpress/MIAMExpress (**http://www.ebi.ac.uk/arrayexpress/**, **http://www.ebi.ac.uk/miamexpress/**) (OrganismPart); microarray data at the MRC Rosalind Franklin Centre for Genomics Research (**http://www.hgmp.mrc.ac.uk/Registered/Menu/microarrays.html**) (mouse and human only); Stanford Microarray Database (**http://genome-www5.stanford.edu**) (free text microarray sample annotations mined for terms); GXD (for blot and cDNA data only); RAD (microarray data). Overall, the SAEL matched 80% of terms in these annotations. We are therefore confident that its use will cover the annotation of a large fraction of sample-based data. We will continue to refine and extend the SAEL in response to the requirements of the bio-ontologies and research communities. For example, it is clear that there are advantages to be gained by relating gross anatomical terms to terms denoting cell types and by relating embryological anatomy to developmental stage.

The principal aim of the SAEL is to fulfil the practical purposes described in the introduction. In relation to wider goals, it is clear that the 'integration' achieved by the SAEL is rather superficial with respect to the very significant differences in concept and purpose between anatomical ontologies. However, by focusing attention on the definitions and relationships of the same high-level terms in different ontologies, it may provide a useful step towards deeper integration. The approach taken in developing the SAEL brings complex and growing anatomy ontologies into the functional genomics domain. As ontologies grow larger, they become more difficult to handle in applications and a detailed knowledge is required to use them. A similar approach has been taken by the Gene Ontology consortium, who provide GO slims. GO slims provide a high level view of the ontologies and are used in applications such as the GO slim tool provided by SGD (**http://www.yeastgenome.org/help/goslimhelp.html**), which allows mapping of the granular GO annotations of a query set of genes to one or more high-level, parent GO Slim terms for a given species. One can imagine a use case where a set of gene products are mapped to GO terms using such a tool, and where complex anatomical annotation from source anatomy ontologies for the same set of query genes are mapped to high-level concepts, such as 'heart'. Combinatorial use of such high-level ontologies allows first-pass analysis of gene function and provides anatomical information for high-throughput data. Further analysis can then be performed where gene products of interest are identified.

### Acknowledgements

We are grateful to the MRC Human Genetics Unit for hosting and partly funding the SOFG Workshop in April 2004. The Workshop was organized by Albert Burger, Jonathan Bard and Duncan Davidson. The text of this paper is based on a presentation given by Helen Parkinson at the ISMB Bio-ontologies meeting in Glasgow, 30 July 2004.

### References

Adult Mouse Anatomical Dictionary: **http://www.informatics.jax.org/searches/AMA_form.shtml**

Copyright © 2004 John Wiley & Sons, Ltd. 

ArrayExpress: **http://www.ebi.ac.uk/arrayexpress/**

Bard JBL, Kaufman MH, Dubreuil C, *et al*. 1998. An internet-accessible database of mouse developmental anatomy based on a systematic nomenclature. *Mech Dev* **74**: 111–120.

Brazma A, *et al*. 2003. ArrayExpress — a public repository for microarray gene expression data at the EBI. *Nucleic Acids Res* **31**: 68–71.

CIBL: **http://www.cbil.upenn.edu/anatomy.php3**

EMAGE: **http://genex.hgu.mrc.ac.uk**

EMAP: **http://genex.hgu.mrc.ac.uk/Databases/Anatomy/**

FMA: **http://fma.biostr.washington.edu/**

GALEN: **www.opengalen.org**

GXD: **http://www.informatics.jax.org/mgihome/GXD/about GXD.shtml**

Hall BK. 1995. Homology and embryonic development. In *Evolutionary Biology*, Hecht MK, *et al*. (eds). Plenum: New York; **28**, 1–37.

Hill DP, Begley DA, Finger JH, *et al*. 2004. The Mouse Gene Expression Database (GXD): updates and enhancements. *Nucleic Acids Res* **32**: (database issue): D568–571.

Hunter A, Kaufman MH, McKay A, *et al*. 2003. An ontology of human developmental anatomy. *J Anat* **203**: 347–355.

Manduchi E, Grant GR, He H, *et al*. 2004. RAD and the RAD Study-Annotator: an approach to collection, organization and exchange of all relevant information for high-throughput gene expression studies. *Bioinformatics* **20**: 452–459.

MGED: **http://mged.sourceforge.net/ontologies/index.php**

MIAMExpress: **http://www.ebi.ac.uk/miamexpress/**

MRC Rosalind Franklin Centre for Genomics Research Microarray database: **http://www.hgmp.mrc.ac.uk/Registered/Menu/microarrays.html**

Rosse C, Mejino JLV Jr. 2003. A reference ontology for bioinformatics: the Foundational Model of Anatomy. *J Biomed Inform* **36**: 478–500.

SOFG: **http://www.sofg.org**

Stanford Microarray Database: **http://genome-www5.stanford.edu**

XSPAN: **http://www.xspan.org**

Copyright © 2004 John Wiley & Sons, Ltd.
